# Key interaction networks: Identifying evolutionarily conserved non‐covalent interaction networks across protein families

**DOI:** 10.1002/pro.4911

**Published:** 2024-02-15

**Authors:** Dariia Yehorova, Rory M. Crean, Peter M. Kasson, Shina C. L. Kamerlin

**Affiliations:** ^1^ School of Chemistry and Biochemistry, Georgia Institute of Technology Atlanta Georgia USA; ^2^ Department of Chemistry—BMC Uppsala University Uppsala Sweden; ^3^ Department of Molecular Physiology University of Virginia Charlottesville Virginia USA; ^4^ Department Biomedical Engineering University of Virginia Charlottesville Virginia USA; ^5^ Department of Cell and Molecular Biology Uppsala University Uppsala Sweden

**Keywords:** protein interaction networks, class A β‐lactamases, protein contact analysis, protein engineering

## Abstract

Protein structure (and thus function) is dictated by non‐covalent interaction networks. These can be highly evolutionarily conserved across protein families, the members of which can diverge in sequence and evolutionary history. Here we present KIN, a tool to identify and analyze conserved non‐covalent interaction networks across evolutionarily related groups of proteins. KIN is available for download under a GNU General Public License, version 2, from https://www.github.com/kamerlinlab/KIN. KIN can operate on experimentally determined structures, predicted structures, or molecular dynamics trajectories, providing insight into both conserved and missing interactions across evolutionarily related proteins. This provides useful insight both into protein evolution, as well as a tool that can be exploited for protein engineering efforts. As a showcase system, we demonstrate applications of this tool to understanding the evolutionary‐relevant conserved interaction networks across the class A β‐lactamases.

## 1 INTRODUCTION

Non‐covalent interactions between residues play a critical role in determining protein structure and function. Within families of evolutionarily related proteins, studying these interactions can provide insights into functional specificity and evolutionary constraints placed on the sequences (Jack et al., 2016). However, considering the large number of non‐covalent interactions in any given protein, traditional experimental techniques often lack the high‐throughput capabilities or resolution required for a comprehensive analysis of these relationships. Computational sequence‐based approaches such as EVcouplings (Hopf et al., 2019), GREMLIN (Kamisetty et al. 2013; Ovchinnikov et al., 2014), Protein Sparse InverseCOVariance (PSICOV; Jones et al., 2012), Direct Coupling analysis (DCA; Morcos et al., 2011) have been applied to address this gap. These techniques rely on a large, diverse sample of available sequences and are thus less suitable for characterizing small groups of evolutionarily related proteins. Structural information, particularly those based on residue interaction networks (RINs), can provide an alternative way to identify preserved interaction motifs and co‐evolving groups of residues. A RIN is a graph‐based representation of a protein structure where nodes represent individual residues and edges represent physico‐chemical interactions between them (Clementel et al., 2022). Combined with graph‐theoretic analysis techniques, RIN‐based tools have demonstrated success in analysis of stability, folding, and function of proteins (Atilgan et al., 2004; Hu et al., 2007; Tse & Verkhivker, 2015).

In this study, we introduce a tool named Key Interaction Networks (KIN), a Python package that can construct a conservation‐based RIN for a set of evolutionary related proteins. While there already exist a number of tools for analyzing protein interactions in multi‐state structures (del Conte et al., 2023; Huggins et al., 2018; Sladek et al., 2021), KIN provides the additional capability to analyze any group of related proteins by finding a common network of interactions that are conserved within the family. Specifically, using KIN, the RIN across the protein family can be projected onto a structure of interest and used to identify both conserved and variable interaction subnetworks throughout the family. Further, the RINs used can be determined directly from analysis of a single structure of each member (e.g., a crystal structure) or alternatively from an ensemble of structures (e.g., molecular dynamics trajectories of simulations performed on each member). To demonstrate the utility of KIN, we applied it to study 69 evolutionarily related class A β‐lactamases, proteins that play a major role in bacterial resistance to β‐lactam antibiotics (Bush & Bradford, 2016). As we will showcase here, projecting conserved interactions onto a structure of interest allows characterization of the core interaction network across a family, which can in turn provide insight into functionally important residues and interactions as well as help to identify relevant residues that could be targeted for mutation. This makes KIN a useful tool both for advancing our understanding of the fundamental biochemistry of enzyme families as well as for protein engineering efforts.

### 1.1 Description of KIN workflow

Residue contact networks have been argued to be powerful tools for modeling evolutionary structural changes in proteins (Zhang et al., 2013). Key Interactions Network (KIN) is an open‐source Python‐based semi‐automated tool that identifies of shared residue interaction networks (RINs) within a community of evolutionarily related proteins. KIN is available for download at https://www.github.com/kamerlinlab/KIN. Prior study in this space has focused on the evolution of interaction networks between proteins and/or other large networks (e.g., Alhindi et al., 2017; Ali & Deane, 2020; Fraser et al., 2002; Levy & Pereira‐Leal, 2008; Pawlowski et al., 2013; Schoenrock et al., 2017; Schüler & Bornberg‐Bauer, 2011; Stumpf et al., 2007; Sun & Kim, 2011; Wagner, 2001; Zitnik et al., 2019, among many others), or on characterizing functionally/allosterically important interaction networks within individual proteins (i.e., without broader evolutionary mapping, e.g., Clementel et al., 2022; Ali & Deane, 2020; Amaro et al., 2007; Brown et al., 2017; Crean et al., 2023; Felline et al., 2022; La Sala et al., 2023; McCormick et al., 2021; Reynolds et al., 2011; Scheurer et al., 2018; Seeber et al., 2011), or has been focused on analysis at the sequence rather than structural level (Anishchenko et al., 2017; Green et al., 2021; Hopf et al., 2019; Lee et al., 2008). KIN focuses on mapping conserved interaction networks within evolutionarily related proteins, which can in turn be responsible for the functionally relevant properties of these proteins such as protein folding stability, allostery or catalysis.

**FIGURE 1:**
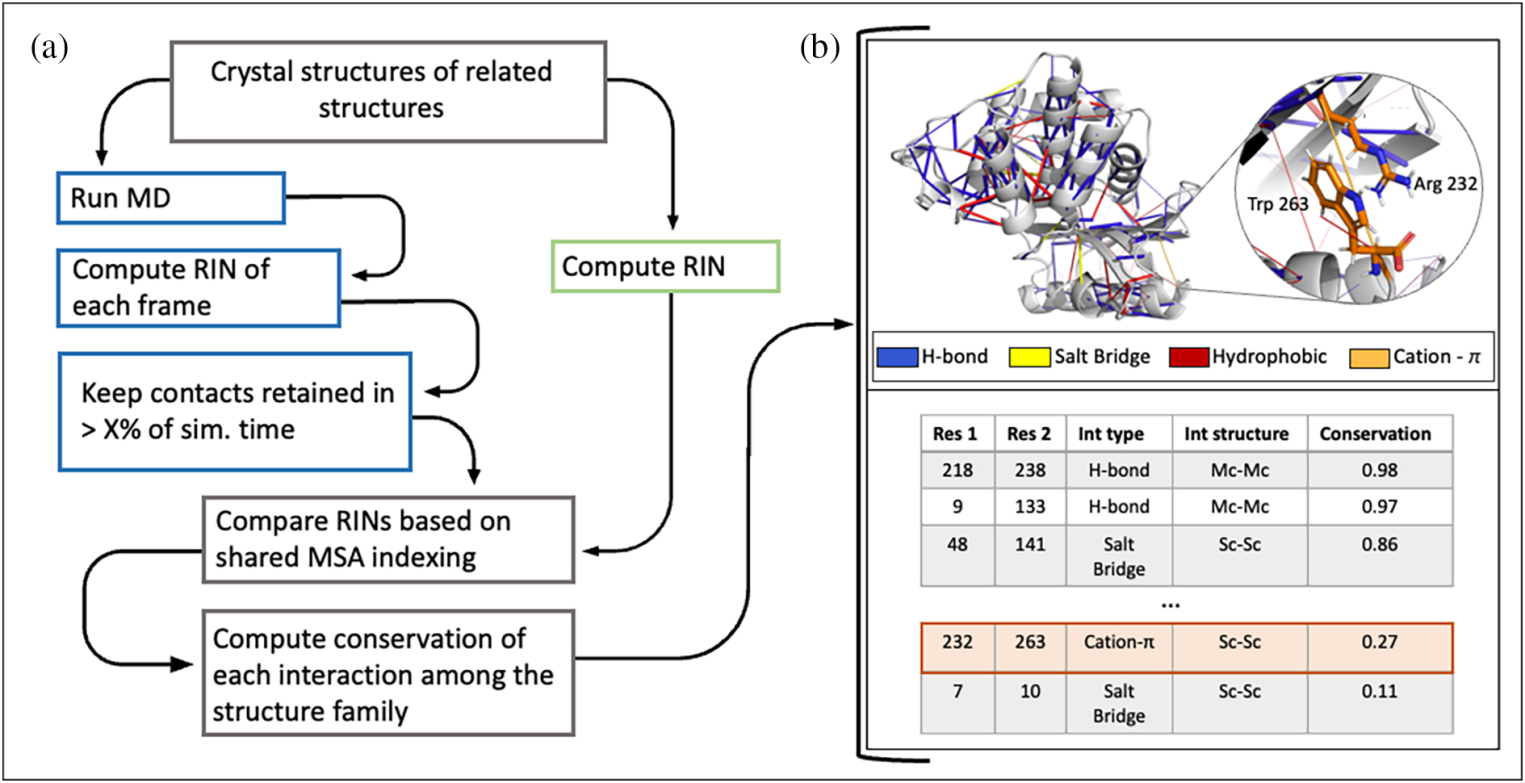
(a) An overview of the main steps in the Key Interaction Networks (KIN) workflow. (b) Example of two output formats of the method: (top) Structural projection of interactions, where lines indicate a contact between two residues. The color of the line reflects the interaction type while the thickness is proportional to the level of conservation. (bottom) Summary of the interaction type, type of interacting residues (main‐chain, Mc, or side‐chain, Sc) and conservation scores for a subset of the interactions preserved in the protein family of interest.

The general workflow of KIN is schematized in Figure 1a and described below. After identifying a group of related proteins to study, all PDB files are downloaded and prepared for analysis. Preparation involves adding missing atoms/residues; assigning protonation states; and standardizing the format of the PDB files to that used by Amber (see Data S1). This file format standardization generates consistent residue and atom labelling schemes that facilitate comparison across related proteins. At this point, KIN analysis can be performed in one of two ways: either on the PDB structures directly, or alternately, biomolecular simulations can be run using AmberMD and the resulting trajectories analyzed with KIN. As alluded to in Figure 1a, RINs can then be constructed based on either structural or simulation data, with non‐covalent interactions classified into any of the following types: salt bridge, hydrogen bond, cation‐π, π‐π, hydrophobic and van der Waals (vdWs) interactions. If MD simulations are performed to generate a structural ensemble for each protein, then KIN uses an adjustable cutoff to determine how frequently an interaction must be present for inclusion in that protein's RIN. For example, if the cutoff is set to 10%, contacts will be included if present for at least 10% of the simulation time.

However generated, the resulting RIN graphs are converted to a multiple sequence alignment (MSA)‐based indexing representation for conservation analysis. The MSA is created using Modeller (Šali & Blundell, 1993) and uses the residue type as the alignment feature (see the Data S1 for more information). At this point, an appropriate reference structure is selected to project the results onto. Conservation scores are computed for each interaction possible within the target protein. This score can be computed in two alternative ways, either the ratio of the number of proteins where a contact is present divided by a total number of proteins or the ratio of the number of proteins where a contact is present divided by the number of proteins that contain the interacting residues at appropriate MSA positions. The second approach means that the conservation score is not penalized if it would be impossible for some structures to form certain interactions because either or both residues involved are not present. These two approaches are designed to account for structural diversity of a family. While many commonly studied families exhibit high structural homogeneity, intriguing mechanisms such as enzyme promiscuity and gene fusion are often observed in in families and superfamilies with a high level of diversity (Das et al., 2015). In this study, we perform all calculations with the first method, as the two results are nearly identical for a family of similar structures.

Additionally, conservation scores of the residue pairs can be summed into per residue scores and normalized using Min‐Max scaling. This is defined as follows, where ***N*** is the total number of contacts that include residue ***r***_***i***_ and ***S*** is the distribution of all unnormalized per residue scores ***s***:

(1) $$s\left(r_{i}\right)=\sum_{j}^{N}{\left(c\text{onservation score}\right)}_{j}$$

(2) $$S=\frac{s-\min\left(s\right)}{\max\left(s\right)-\min\left(s\right)}$$

The conservation scores for each contact can be visualized on a structure of choice using the PyMOL scripts generated by KIN or analyzed further in tabular format (Figure 1b). The tabular dataset is stored as a pandas (McKinney, 2010; The Pandas Development Team, 2020) DataFrame, with each row defining a different contact. Columns include: residue numbers (with both MSA and PDB numbering); interaction type (e.g., hydrogen bond); which parts of each residue are involved in the contact (i.e., side or main chain); and the conservation score. All of these columns can easily be filtered for further analysis, such as to highlight only the most conserved interactions across a family of proteins.

**FIGURE 2:**
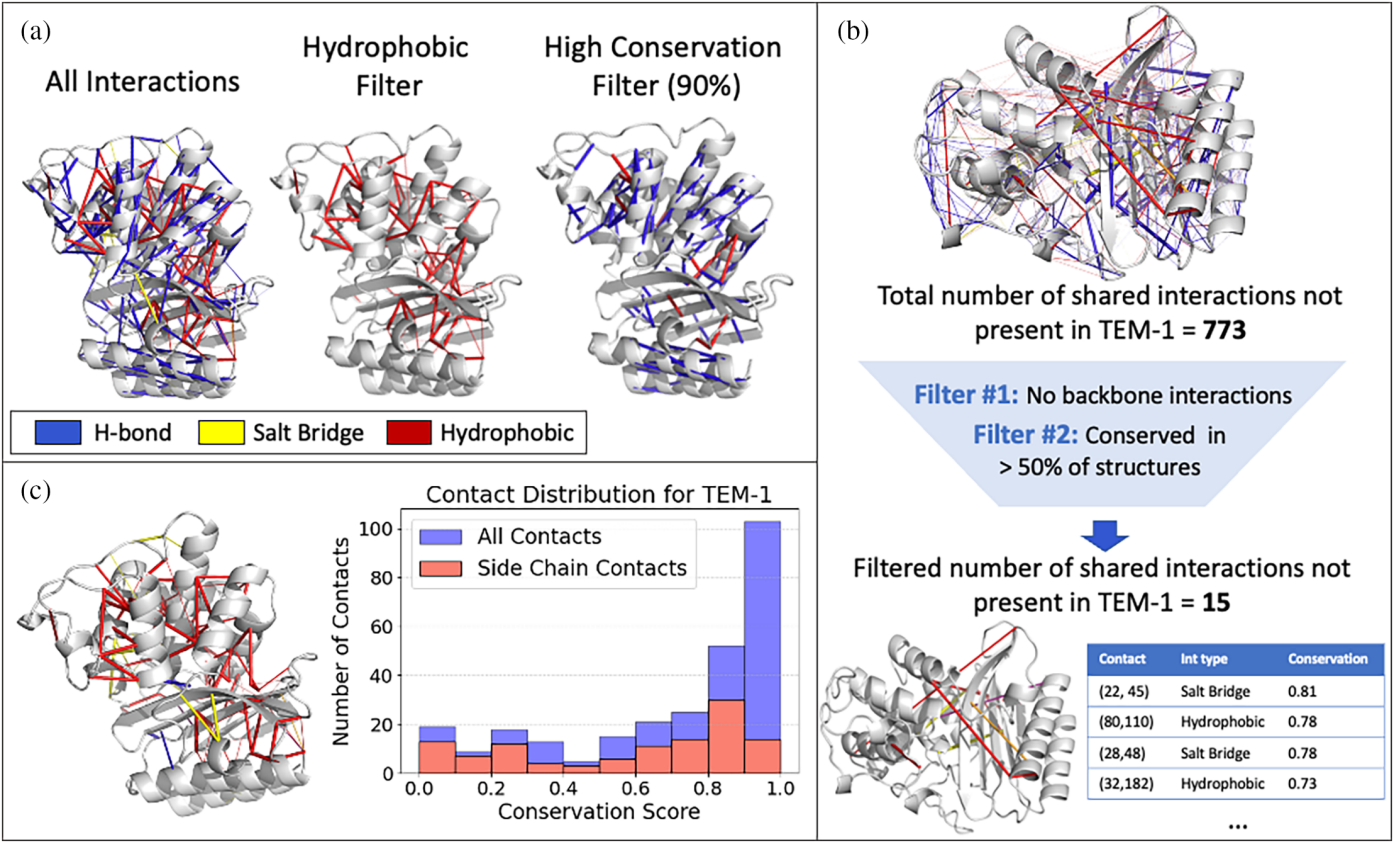
(a) The conserved interaction network of the class A β‐lactamase family is projected onto the structure of TEM‐1 (PDB ID: 1M40; Minasov et al., 2002). Data are shown with no filtering applied (“all interactions”), only hydrophobic interactions selected (“hydrophobic filter”), and only those interactions present in 90% or more of structures selected (“high conservation filter”). (b) “Missing” interactions are rendered, defined as interactions present in >50% of class A β‐lactamase proteins but not present in TEM‐1. This also gives an example of consecutive filtering. (c) Conserved interactions involving at least one side‐chain are rendered. Also shown is histogram of contacts versus conservation scores showing both all contacts and side‐chain‐only contacts.

To illustrate this and further capabilities of KIN, we used this tool to find shared interaction networks among 69 structures of class A β‐lactamases. We projected these interaction networks onto the structure of TEM‐1 (PDB ID: 1M40; Minasov et al., 2002) as a representative system throughout, as this enzyme is commonly associated with clinical ß‐lactam resistance in Gram‐negative bacteria (Bradford, 2001; Livermore, 1995) and is considered a model class A β‐lactamase (Brown et al., 2009). The most conserved interactions among β‐lactamases (Figure 2a) comprise hydrogen bonding networks within secondary structural elements and highly preserved hydrophobic core interactions. Interactions can also be filtered by whether the side chain or main chain is involved, and filters can be applied consecutively to focus on a specific set of interactions more deeply (Figure 2b). Users can also obtain the set of interactions conserved within the analyzed family but not present in the protein of interest (Figure 2c). This is designed to help understand why a particular protein differs functionally from the rest of an evolutionary group or potentially to guide protein engineering.

### 1.2 Comparison of static and dynamic KIN analysis

**FIGURE 3:**
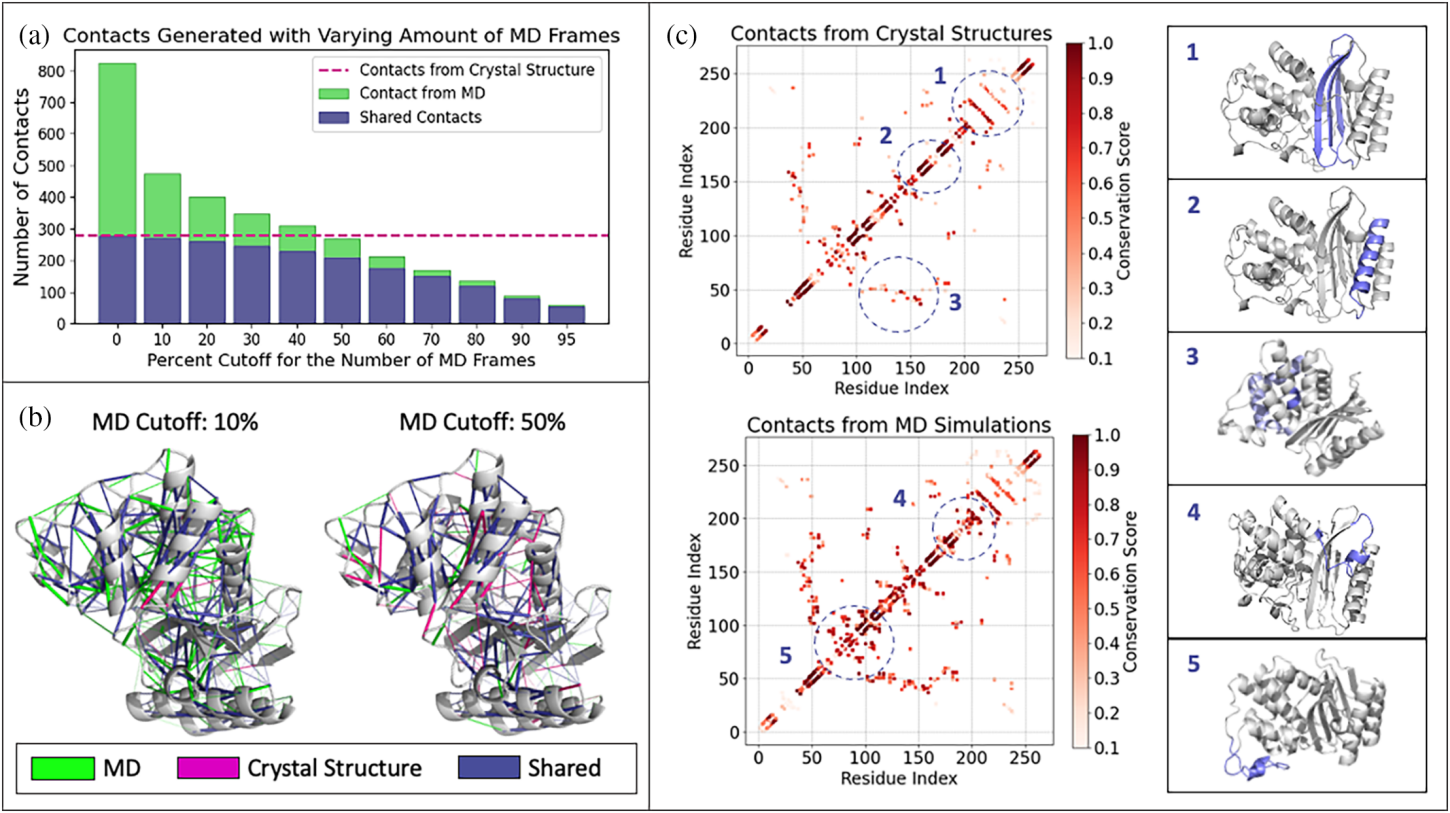
(a) Comparison of the number of contacts identified with different MD retention cutoffs used, defined as the minimum percentage of MD frames in which an interaction must be present to be included. The number of contacts found by analyzing PDB structures alone (red dotted line) is shown for reference. Shared contacts are contacts found in both crystallographic and MD‐derived networks. (b) Visual comparison of the interaction networks calculated from MD trajectories using 10 and 50% cutoffs. Shared interactions are defined as interactions present in both MD and crystallographic networks. (c) Contact maps for (top) crystallographic and (bottom) MD RIN of 10%. These data show a high matrix correlation, with a Pearson coefficient of 0.86. Structural elements associated with the interaction pattern reflected on the contact maps are shown in subfigures (Hopf et al., 2019; Jack et al., 2016; Jones et al., 2012; Ovchinnikov et al., 2014) and highlighted in purple.

For comparative purposes, we performed KIN analysis on both static (crystallographic) structures available in the Protein Data Bank (Ovchinnikov et al., 2014) and on molecular dynamics trajectories of the respective structures. MD simulations of all 69 structures were performed and prepared according to the protocol described in the Data S1. A tunable cutoff parameter specifies what fraction of the simulation frames an interaction must be present in order to be counted for that protein. Figure 3a shows the dependence of interactions detected on cutoff value, compared to analysis of PDB structures only. We will refer to this reference analysis of PDB structures as the crystallographic network. As might be expected, including all interactions present in any frames (cutoff >0%) of the simulation is highly sensitive but skewed by rare events. Setting the cutoff to even ≥10% still captures almost all of the crystallographic network while reducing the number of transient interactions (Figure 3a). Such transient interactions may be functionally important or may be artifactual, which is why a user‐tunable parameter is critical here. Increasing the cutoff values between 10 and 50% results in a network more similar to the crystallographic network, with the number of shared residues between the two networks decreasing a lot slower than the number of the MD contacts (Figure 3a). This means that the interactions removed as a result of the increasing cutoff are largely non‐crystallographic.

A visual comparison of the conserved interaction network obtained from MD simulations using per‐trajectory cutoffs is rendered in Figure 3b. The majority of interactions that are preserved between crystallographic and MD‐based networks (blue) describe interactions within secondary structure components. As might be expected, interactions found only via MD analysis are located primarily in the more flexible regions of the system. It is important to note that many of the dynamic interactions that are discarded when using a higher cutoff are strongly conserved across class A ß‐lactamases and thus may contain important information (Figure S1). For example, we observe that, of the interactions preserved when a per‐trajectory cutoff of 10% is used instead of 50%, 32% (47 out of 143) are conserved in >80% of ß‐lactamase proteins analyzed. Further, while a number of hydrogen bonding interactions dominate both MD‐based and crystallographic networks, cutoff sensitivity analysis shows many hydrophobic interactions to generally be more transient (Figure S2). Many of these interactions are not present in the crystallographic network and yet are highly conserved across the family.

Reside contact maps provide another means to compare crystallographic and MD‐based interaction networks (Figure 3c). Here, we plot crystallographic contact maps and MD‐based ones analyzed using a 10% cutoff. Generally, interactions within α‐helices and β‐sheets are constant between the two. In contrast, more dynamic regions displayed greater variation in the contacts found and their relative conservation scores, particularly interactions between neighboring α‐helicies or within loops and linker regions. These observations support the notion that the conserved interactions within secondary structural elements can be reliably described with either of the methods, but the analysis of crystallographic structures alone might be insufficient to capture more dynamic interactions.

### 1.3 Conservation of interaction networks in the β‐lactamase family

In the following section, we use the set of class A β‐lactamase structures to illustrate further system‐specific analysis that can be done using KIN. The conserved interaction network derived from crystallographic data is rendered in Figure S2 and clearly shows that most of the highly conserved interactions are inter‐molecular hydrogen bonds within α‐helices or hydrophobic contacts in the core of the protein. When the contact maps are plotted by interaction type, hydrogen bonding interactions clearly predominate, while dense interactions around residue 50 characterize the hydrophobic core (Figure S3). We performed a structural alignment of all 69 class A β‐lactamases (Figure S4) to the TEM‐1 structure and observed the α‐helices and the core of each protein to be structurally preserved among the majority of these structures with RMSD score of 0.42 Å, and are an important common feature across the β‐lactamase fold (Philippon et al., 2015; Tooke, Hinchliffe, et al., 2019b).

**FIGURE 4:**
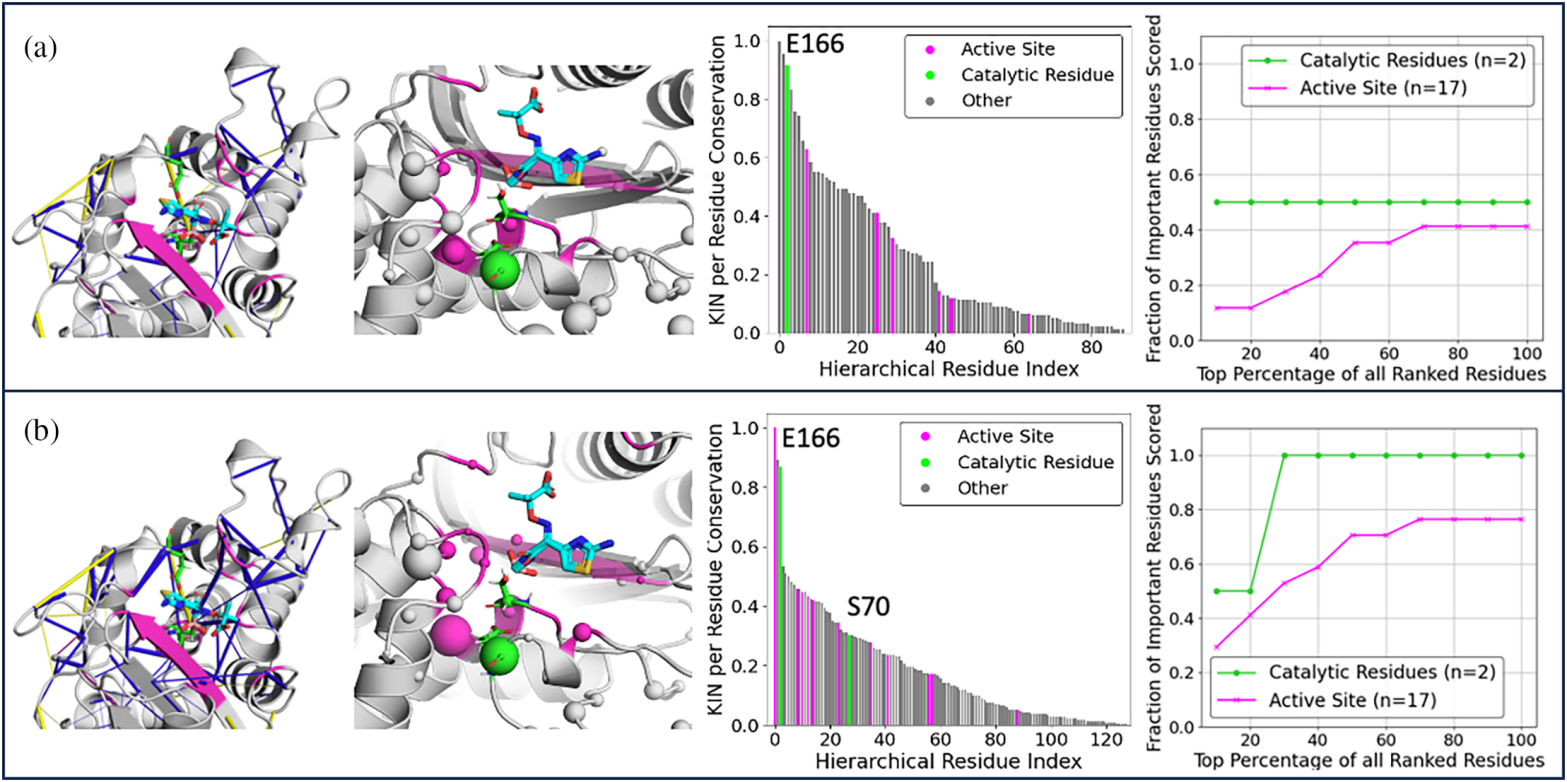
Analysis of the conserved interaction network at the TEM‐1 active site obtained from KIN analysis of (a) crystallographic data and (b) molecular dynamics (MD) trajectories. Residues that are directly involved in the catalytic mechanism are colored green, those that form the active site are colored purple, and all other residues are colored gray. The interactions analyzed were filtered to only include non‐hydrophobic interactions involving at least one side‐chain. These conserved interactions are visualized in the left panel, with relative conservation of the interaction indicated by cylinder size. The middle‐left panels are projections of the per‐residue interaction conservation score, with each residue's C_α_‐atom shown as spheres (relative conservation indicated by sphere size). The middle‐right panels display hierarchically ordered KIN per residue scores with catalytic and active site residues highlighted. The right panels display an analogue to the receiver operator characteristic where the fraction of target residues identified is plotted as a function of the residue rank based on a summed per‐residue conservation score. Protein structures are based on PDB ID: 1M40 (Minasov et al., 2002), with the co‐crystallized transition state analogue (light blue) shown for reference. A 20% MD simulation retention cutoff was used for analysis.

We also explored the relationship between conserved interaction networks and the active site architecture (Figure 4). Each residue was classified as “Catalytic” (directly involved in catalysis), “Active Site” (within 5 Å of a co‐crystalized transition state analogue, see Figure 4) and “Other” (all other residues). (Jelsch et al., 1993; Minasov et al., 2002). Because we are interested in polar interactions that might vary with mutation, we filtered the interactions to only include those that were non‐hydrophobic and involved at least one side‐chain residue. This decision was based on the observations made from Figure S4, in which the most strongly conserved interactions formed across the β‐lactamases are hydrogen bonds between two main chain residues inside the α‐helices and hydrophobic interactions at the core of protein. These interactions were filtered out in order to not dominate the subsequent analysis in Figure 4.

Both our analysis on the crystal structures and that on the MD simulation dataset highlight E166 (which acts as a general base in class A β‐lactamase catalysis; Kalp et al., 2009) to be one of the residues with the highest per residue conservation score. This is primarily as a result of the highly conserved hydrogen bonds E166 forms with the side‐chains of residues S70, N136, and N170, and the salt bridge with K73. Taken together, this would suggest these interactions with E166 are essential for class A β‐lactamase catalysis, which is consistent with the literature on class A ß‐lactamases (Ji & Boxer, 2022; Kalp et al., 2009). However, only the MD‐based network is able to identify conserved interactions for the second catalytically important residue, S70. This residue is responsible for the formation of a covalent acyl intermediate during the catalytic reaction (Padayatti et al., 2004). MD simulations sampled alternative S70 conformations that were not observed in available crystal structures. Subsequent KIN analysis identified conserved hydrogen bonds between S70–K73 and S70–E166. These interactions are consistent with the established role of S70 in facilitating proton transfer between K73 and E166 (Cs et al., 2019; Meroueh et al., 2005). Additionally, Figure 4 illustrates that analysis of MD trajectories gave more insight into highly conserved interactions concentrated around the active site. The MD‐based network identifies conserved interactions involving 78% of the active site residues, while the crystallographic network identifies only 41%. Furthermore, 40% of the MD‐identified residues are contained within the top 20% of the KIN per‐residue scores. These conserved interactions within the active site region form a hydrogen‐bonding network. These observations demonstrate that KIN can serve as a useful complementary tool for characterizing evolutionarily conserved active site interactions.

### 1.4 Comparison of KIN to sequence based data

Contact‐based and sequence‐based analyses of protein evolutionary groups are expected to be complementary and to some extend concordant. One such way to compare the similarity of two proteins at the sequence level is to calculate their percentage identity, or what fraction of amino acids in an aligned sequence are identical. The percentage identity matrix (PIM) for all ß‐lactamases studied is shown in Figure S5A. As a means of comparison, we also generated a protein contact similarity matrix (PCSM) based on contact networks (Figure S5B), with the metric the ratio of conserved contacts against non‐conserved contacts between any two proteins. The Spearman rank order correlation between the PIM and PCSM matrices is 0.71. This indicates relatively strong concordance between evolutionary conservation of key interaction networks and sequence conservation across class A β‐lactamases.

**FIGURE 5:**
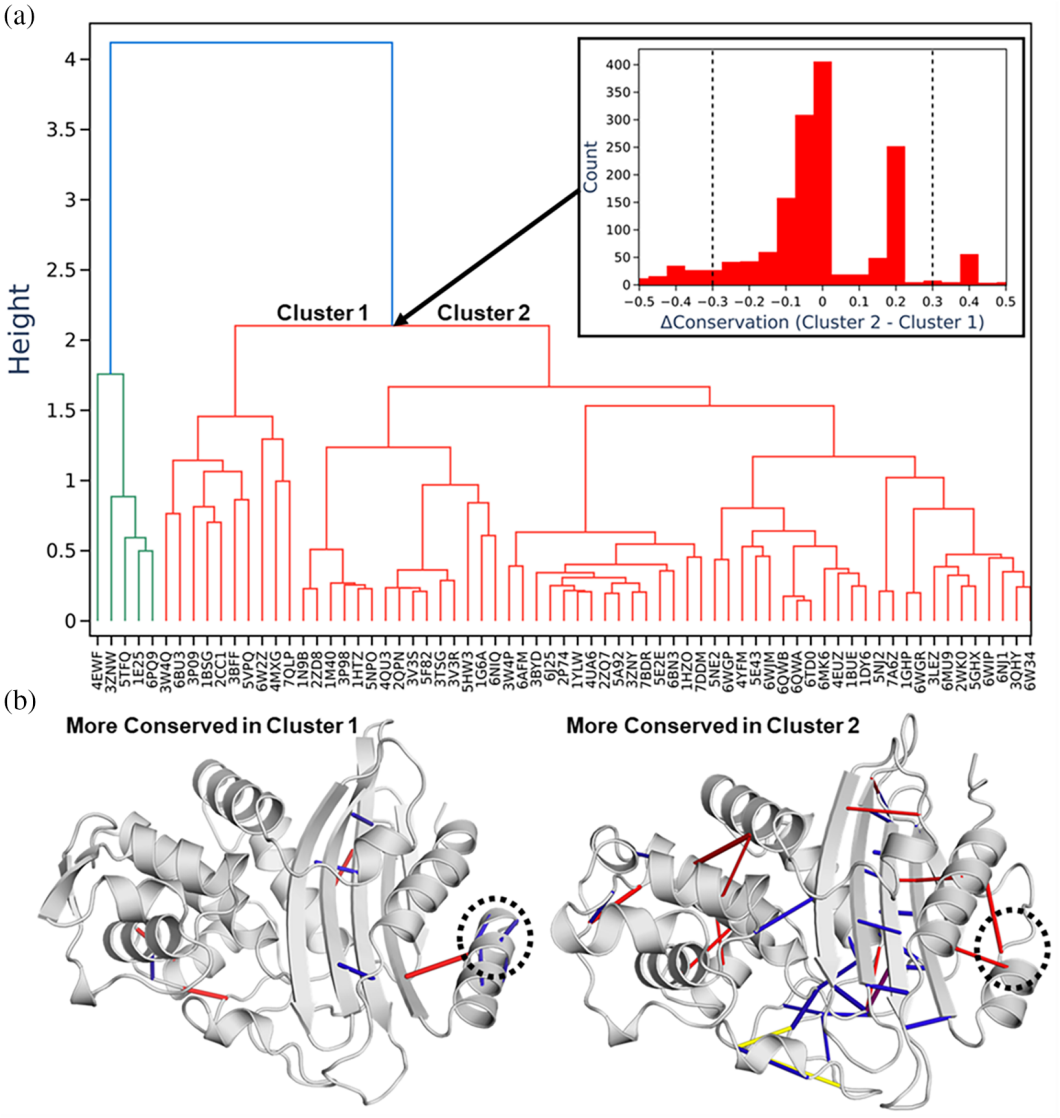
(a) Hierarchal clustering (average linkage) performed on 69 β‐lactamase crystal structures using the ratio of shared and unshared contacts between each protein structure pair to create a similarity matrix as input for the clustering. Each β‐lactamase is labeled according to its PDB ID (Berman et al., 2000). A split in the structures was selected to divide the β‐lactamases into two groups (Clusters 1 and 2). The panel inset shows the difference in conservation for each contact between the two clusters. The two vertical dotted lines at ±0.3 indicate the cutoffs used to select which contacts to present in “B.” (b) Projection of the largest differences in contact conservation onto representative crystal structures from Clusters 1 (class A β‐lactamase from *Mcyobacterium fortitum*, PDB ID: 2CC1; Sauvage et al., 2006) and 2 (KPC‐4, PDB ID: 6QWB; Tooke, Hinchcliffe, et al., 2019a) of the dendrogram, respectively. A region of each protein with a relatively large difference in the contact network is circled.

The PCSM also enables hierarchal clustering to generate a contact‐based equivalent of a phylogenetic tree (i.e., a dendrogram, Figure 5a), using a distance measure based on how similar their interaction networks are. Analyzing the interactions that differentiate two sub‐trees can yield insight into putative evolutionary or functional differences. As an example of this, we selected a branch point in the dendrogram and used this to create two groups of proteins, labeled Clusters 1 and 2 in Figure 5a. Each contact was scored by the difference in degree of conservation between the two clusters, quantified as C_2,*ij*_–C_1,*ij*_, where C_1,*ij*_ is the fraction of structures in group 1 where contact *ij* is present, and the resulting histogram is plotted in Figure 5a. This histogram clearly shows most contacts do not notably differ between the two clusters. To study those contacts that differ the most between the two clusters, we selected contacts with a conservation difference of ≥ ± 0.3. Of the 58 contacts thus selected (from 1712 total), those with the greatest difference in contact conservation are rendered in Figure 5b, on the structure of a representative protein from each cluster.

While a detailed analysis of the differences in the contact networks of β‐lactamases is beyond the scope of this manuscript, as an example, our comparison of Clusters 1 and 2 identified several well conserved intermolecular hydrogen bonds towards the N‐terminus of the Cluster 1 β‐lactamases (circled on Figure 5b) resulting in an extended α‐helix for the Cluster 1 β‐lactamases.

We identified the phylum to which each β‐lactamase belonged in order to compare contact based hierarchal clustering against taxonomic classification (Figure S6). While there clearly was some level of cluster separation according to the phylum for example 9/10 of the *Bacillota* were clustered to the far right of the dendrogram, it could not explain all of the separations (Figure S6). The most striking example of this is the most distinct cluster found in the dendrogram which is separation that occurs at a height of ~4 (Figure 5) and is made up of five β‐lactamases. These five β‐lactamases contain three of the six phyla present throughout the whole dataset (Figure S7). We applied the same approach as described for Figure 5b to identify which contacts differed between the two groups, which by and large showed hydrogen bonds distributed throughout most of the protein to differ (Figure S8).

Finally, we note that an alternative strategy could be to first construct a phylogenetic tree/dendrogram using sequence data (which would contain many more examples), but then interpret a branch point of interest in the phylogenetic tree using the approach we described above based on available crystal structures and/or protein structure prediction.

### 1.5 Applicability of KIN towards protein engineering

Many existing protein engineering tools such as dTERMen (Zhou et al., 2019), FuncLib (Khersonsky et al., 2018) and Hotspot Wizard (Sumbalova et al., 2018) make use of sequence‐based information from homologues to help select candidate mutations. These tools hypothesize that mutating a residue to an amino acid that is found in homologous proteins at that same position is less likely to give rise to a “harmful” mutation (Frappier & Keating, 2021). The information generated from our conservation network analysis allows for an implementation of the same idea, and in the following section, we will demonstrate how this could be applied using TEM1 as an example, as a potential first step in a protein engineering pipeline.

**FIGURE 6:**
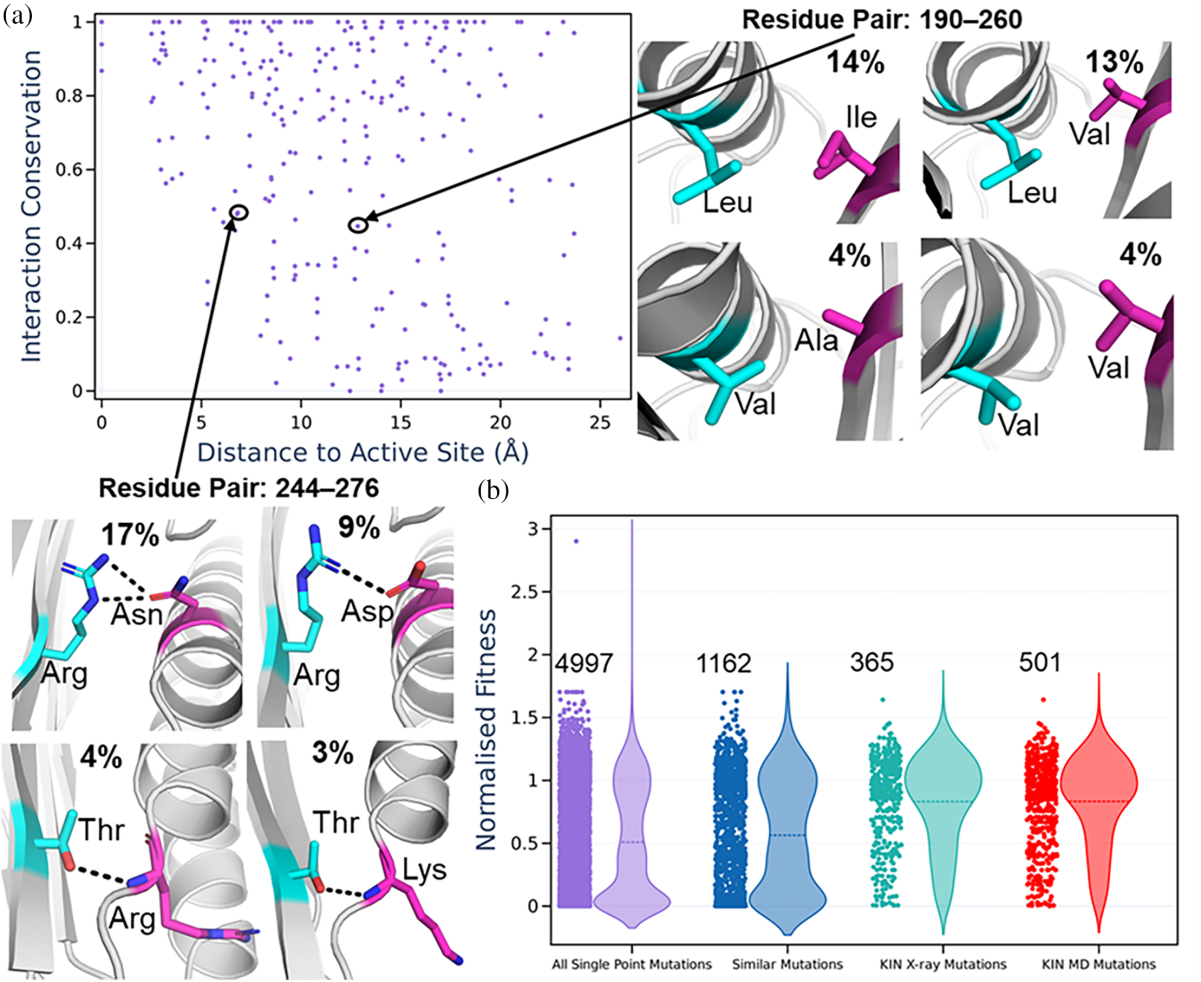
(a) Scatter plot showing each interacting/contacting residue pair in TEM1, with the contact conservation shown against its distance from the TEM1 active site. Two contacts on the plot are highlighted, with the four most common interacting residue pairs in our dataset of 69 structures shown in both cases. (b) A strip and violin plot of the normalized fitness values of four different selections of single point mutations for TEM1. The fitness is a measure of a given proteins ampicillin resistance and is normalized such that the WT has a fitness of 1. For each violin plot, the mean fitness is depicted as a dotted line. The first selection is simply all possible single point mutations (that were measured by Firnberg et al. (2014). The second selection is made up of single point mutations that exchange mutations to similar residues (e.g., Leu to Val, Arg to Lys), see  S1 for further details. The third selection is all possible single point mutations obtained from our approach (KIN) using just data from the X‐ray crystal structures. The final selection uses MD‐based contact networks to identify potential mutations.

Using the crystal structure of TEM1, we defined the distance that each interacting pair of residues is from the active site and plotted this against the conservation of the contact score (Figure 6a). This enabled us to identify contacts distributed throughout TEM1 that are both proximal and distant from the active site, with varying levels of conservation. Figure 6a shows two examples of such contacting pairs in TEM1, with one example involving two pairs of variable hydrophobic residues forming hydrophobic contacts within the core. While in TEM1, the residues that provide this hydrophobic interaction are Leu and Ile, we can identify the other combinations of residue pairs that form this interaction, which would require a single or double mutation to occur in TEM1. In comparison to the hydrophobic interaction described above, our second example shows a hydrogen bond/salt bridge interaction between the residues Arg and Gln in TEM1. Interestingly, two of these alternate interactions show a rather substantial conformational difference in which one of the helices is now a loop, and the interaction formed is now between the sidechain and the backbone instead of between the two sidechains (Figure 6a).

The approach described above provides a candidate set of 365 single point and 982 double point mutations that could be considered for protein engineering purposes, as opposed to the ~5000 possible single and ~25,000,000 possible double mutations from the 263 residues of TEM1. Given that these mutations are observed in other related lactamases, these mutations would be expected to be more tolerant of mutation. To evaluate the “usefulness” of this approach, we utilized data generated by Firnberg et al. (2014), in which protein fitness values for ampicillin resistance were determined for all single point mutants of the TEM1 β‐lactamase. We compared the fitness values obtained from our subset of single point mutations against all single point mutations, as well as selecting single point mutations of a similar amino acid type to the wild‐type amino acid (Figure 6b). As expected, selecting only single point mutations similar to the WT amino acid gives rise to a notable improvement, with the mean fitness value changing from 0.51 to 0.57 (as compared to using all possible single point mutations), Figure 6b. By applying KIN and using only the crystal structure contact data we obtained a substantial improvement, reaching a mean fitness value of 0.83, Figure 6b. We also assessed if including interactions from MD‐based contact networks would change the results. Using a per‐trajectory inclusion cutoff of 10%, we obtained 501 candidate single point mutations to test as compared to the 365 from the X‐ray structure alone. As depicted in Figure 6b, there was not an increase in the mean fitness values from the inclusion of MD simulation data. It should be noted that the MD‐based networks include more potential mutations (which could be subsequently filtered and/or combined in subsequent steps), so the denominator to the mean fitness value increases. We also performed a sensitivity analysis with regard to per‐trajectory cutoff score (Figure S9). The shape of the distributions and mean values for the distributions obtained were very similar for both “strict” and “relaxed” cutoffs, with the only notable (and expected) difference being that using a stricter cutoff reduces the number of possible mutations to try.

Finally, one might expect that filtering mutations to only include those within a certain distance from the active site of TEM1 would increase the variance of the selected mutations fitness values. We assessed the impact of filtering mutations using several different distance cutoffs between 5 and 25 Å of TEM1's active site (Figure S10). While there was no notable difference in the distributions of fitness values across the different cutoffs (Figure S10), we caution that this is most probably a system specific effect rather than a general rule.

## 2 DISCUSSION

Protein interaction networks are essential to facilitating protein function, and understanding their evolutionary conservation gives insight into how novel protein functions evolve (Jack et al., 2016). While there exist are a number of tools that can calculate protein interaction networks, including from dynamical trajectories (del Conte et al., 2023; Huggins et al., 2018; Sladek et al., 2021), these typically do not additionally focus on evolutionary conservation, and tools that study evolutionary conservation typically focus on protein–protein interaction networks (Alhindi et al., 2017; Ali & Deane, 2020; Fraser et al., 2002; Levy & Pereira‐Leal, 2008; Pawlowski et al., 2013; Schoenrock et al., 2017; Schüler & Bornberg‐Bauer, 2011; Stumpf et al., 2007; Sun & Kim, 2011; Wagner, 2001; Zitnik et al., 2019) rather than interactions within an individual protein subunit. We note here, however, a recent related study that exploited MD‐based correlation‐based networks in combination with sequence conservation analysis for protein engineering, in order to design new variants of the tryptophan synthase complex using activity enhancing distal mutations (Maria‐Solano et al., 2021).

We present here a new tool, KIN, that is an open‐source Python package that can construct conservation‐based RINs for sets of evolutionary related proteins from both static and dynamic data. Our dynamic approach (MD simulations) was able to identify many novel interactions not observed with the static contact analysis. The current analysis is based on 5 × 100 ns MD simulations per system, which in the case of the current enzymes, proved adequate to provide valuable insight into their contact networks. However, the contacts found are of course modulated by the conformational space explored during the simulation(s), which is an important trade off to consider when using this tool. That is, for more conformationally complex systems, additional replicates or longer simulations may identify more novel contacts, but at the expense of additional computational resources.

We showcase an application of KIN to class A β‐lactamases and demonstrate its usefulness both as a tool to understand protein evolution more broadly, as well as part of the protein engineering toolkit. Our results suggest two possible uses for the KIN tool in protein engineering. The first is to identify sites where point mutagenesis is unfavorable. As shown in Figure 6b, mutation of a single partner in an evolutionarily conserved interaction tends to incur a substantial fitness penalty. These sites should either be spared or co‐mutated so as to maintain the interaction. Second, conserved interactions that are missing from a particular template scaffold are likely good targets for mutational engineering. If these interactions can be reconstituted, we would predict protein stability and/or function to improve, particularly if the template is an ancestral scaffold that may lack some interactions that evolved later.

## Supporting information


**DATA S1.** Supporting information
