# Supplementary material for: Key interaction networks: Identifying evolutionarily conserved non‐covalent interaction networks across protein families
Source: Protein Sci. 2024 Feb 15;33(3):e4911. doi: 10.1002/pro.4911 (PMC10868456; doi:10.1002/pro.4911)
Supplement: Supplementary file 1 — DATA S1. Supporting information [file PRO-33-e4911-s001.pdf]

Supporting Material for:

Key Interaction Networks: Identifying Evolutionarily Conserved Non-Covalent Interaction Networks Across Protein Families

Dariia Yehorova,[1] Rory M. Crean,[2] Peter M. Kasson,[3,4*] and Shina C. L. Kamerlin[1,2*]

1. School of Chemistry and Biochemistry, Georgia Institute of Technology, 901 Atlantic Drive NW, Atlanta, GA 30332, USA.

2. Department of Chemistry – BMC, Uppsala University, BMC Box 576, S-751 23 Uppsala, Sweden.

3. Departments of Molecular Physiology and Biomedical Engineering, University of Virginia, Charlottesville, Virginia 22908, USA.

4. Department of Cell and Molecular Biology, Uppsala University, BMC Box 596, Uppsala 751 24, Sweden.

Corresponding author email addresses: kasson@virginia.edu, skamerlin3@gatech.edu

Table of Contents

S1. Methodology…S3
Structure Selection and Preparation…S3
Structures Selected…S4
Molecular Dynamics Simulations…S4
Contact Analysis…S6
Protein Sequence Analysis…S8
Predicting Candidate Mutations with KIN…S8
S2. Supplementary Figures…S9
S3. Supplementary References…S20

S1. Methodology

Structure Selection and Preparation

The Protein Data Bank (PDB, (1)) IDs for all Class A structures were obtained from the Beta-lactamase database (BLDB) (2) on the 5th of June 2023. This database was then filtered to remove β-lactamases that were synthetic, chimeric or generated from ancestral sequence reconstruction to focus our study on only naturally occurring β-lactamases. In the case of β-lactamases for which more than one structure was available, the structure with the best resolution was selected for download. This resulted in 69 unique class A structures being downloaded from the PDB.

The downloaded structures went through a 5-step procedure in order to prepare them for both crystal structure analysis and molecular dynamics (MD) simulations. This protocol is provided as part of the GitHub repository associated with this publication (https://www.github.com/kamerlinlab/KIN) and described below in brief. (1) Modeller (3) was used to complete missing structural information, resolve any structurally disordered regions, and perform multiple sequence alignment on the full set of monomers. As the class A β-lactamases of interest to this work are monomeric in solution (4; 5), where relevant, we took the first monomeric unit from each crystal structure for our simulations and analysis. This also ensured an equal weighting of each sequence in the subsequent multiple sequence alignment (MSA). (2) MolProbity (6) was then used to determine the optimum tautomerization states for all histidine residues and make any required Asn/Gln side-chain flips under the criteria of optimizing the hydrogen bonding network. (3) The pKa of all residues with more than one possible protonation state was predicted using PROPKA v3.1 (7). If an Asp, Glu or His residue had a predicted $pK_a \geq 8$ or if an Arg, Lys or Tyr had a predicted pKa ≤ 6, they were flagged for visual inspection, and the protonation state was assigned based on examining the local hydrogen bonding network of the residue. (4) The

module “pdb4amber” was run on each structure in order to make each PDB file follow AmberMD formatting. (5) Tleap (8) was used to generate an Amber compatible topology and coordinate file alongside solvate and neutralize each system (for MD simulations). Each structure was described with the Amber ff14SB (9) force field and TIP3P (10) water model. While this workflow is largely automated, some visual examination after step (2) and (3) is required to ensure that the procedure is appropriate for the system of choice. The final structures used for contact analysis are provided in the GitHub repository at https://www.github.com/kamerlinlab/KIN.

Structures Selected

The 69 PDB (1) structures selected for the study on Class A β-lactamases were: 1BSG, 1BUE, 1DY6, 1E25, 1G6A, 1GHP, 1HTZ, 1HZO, 1M40, 1N9B, 1YLW, 2CC1, 2P74, 2QPN, 2WK0, 2ZD8, 2ZQ7, 3BFF, 3BYD, 3LEZ, 3P09, 3P98, 3QHY, 3TSG, 3V3R, 3V3S, 3W4P, 3W4Q, 3ZNW, 3ZNY, 4EUZ, 4EWF, 4MXG, 4QU3, 4UA6, 4YFM, 5A92, 5E2E, 5E43, 5F82, 5GHX, 5HW3, 5NE2, 5NJ2, 5NPO, 5TFQ, 5VPQ, 6AFM, 6BN3, 6BU3, 6J25, 6MK6, 6MU9, 6NIQ, 6NJ1, 6PQ9, 6QWA, 6QWB, 6TD0, 6W2Z, 6W34, 6WGP, 6WGR, 6WIP, 6WJM, 7A6Z, 7BDR, 7DDM, 7QLP. Both the unedited and fully processed PDB files of each structure is provided as part of the GitHub repository: https://www.github.com/kamerlinlab/KIN.

Molecular Dynamics Simulations

Molecular dynamics (MD) simulations were performed using Amber20 (8) on all 69 of the β-lactamase structures prepared above. To prepare each system for production MD simulations, a standard procedure of minimization, heating and equilibration was followed.

For all dynamics steps, a 1 fs timestep was used alongside the SHAKE algorithm (11) to constrain all bonds involving a hydrogen atom. All NVT simulations used Langevin temperature control (collision frequency of 1 ps^{-1}), whilst all NPT simulations used both Langevin temperature control (collision frequency of 1 ps^{-1}) and a Berendsen barostat ((12), 1 ps pressure relaxation time). In order to generate 5 replicas for each system, random velocities were assigned at the heating step (described below) for each system.

The steps were as follows: (1) 1000 steps of steepest descent energy minimization were performed to relax each structure. (2) The system was gradually heated from a starting temperature of 100 K to a final temperature of 300 K over the course of 1 ns. During this heating step, all protein atoms were restrained using 100 kcal mol^{-1} $Å^{-2}$ distance restraints. (3) A 1 ns long NVT simulation was performed with this restraint retained. (4) A 1 ns long NPT simulation was performed but this time with the restraint reduced to 10 kcal mol^{-1} $Å^{-2}$. (5) The restraint was then altered to be on only the backbone heavy atoms of each protein residue and another 1 ns long NPT simulation was run. (6) The restraint magnitude was then reduced to 1 kcal mol^{-1} $Å^{-2}$ for a 1 ns long NPT simulation. (7) The restraint magnitude was then reduced to 0.1 kcal mol^{-1} $Å^{-2}$ for a 1 ns long NPT simulation. (8) Finally, a 1 ns long MD simulation without restraints was performed as the final equilibration step.

For each system, five, 100 ns long replicas of NPT (1 atm and 300 K) production MD simulations were run. Simulations used a timestep of 2 fs with the SHAKE algorithm (11) applied to constrain all bonds to hydrogen. A 10 Å direct space nonbonded cut-off was applied with long-range electrostatics evaluated using the particle mesh Ewald algorithm (13). Temperature and pressure regulation were achieved using Langevin temperature control (collision frequency used was 1 ps^{-1}) and a Berendsen barostat (pressure relaxation time used was 1 ps). Frames were saved

every 100 ps and used as input for contacts calculation. To prepare, for the contact analysis, CPPTRAJ (14) was used to post-process each trajectory (performing both imaging and removing solvent and counter ions).

Contact Analysis

In order to identify the non-covalent protein interactions present in each frame, we combined interaction definitions present in literature within a python module (included as part of the GitHub repository for this package: https://www.github.com/kamerlinlab/KIN) (15; 16). This approach can identify the following interaction types between the 20 standard amino acids: salt bridge; hydrogen bond; cation-*π*; *π*-*π* interaction; hydrophobic and van der Waals (vdWs) interactions (see **Figure S11**) and further specifies if the interaction is from the side or main chain or both parts of the chain. A salt bridge is defined as such if 2 oppositely charged heavy atoms are within 4.5 Å of one another. A hydrogen bond is defined as such if the donor-acceptor distance is ≤ 3.5 Å and the donor-hydrogen-acceptor angle is 180 ± 45°. Definitions for cation-π interactions were taken from Infield *et al.* (16), where a cationic residue is either Arg or Lys and the π-residue is one of Trp, Tyr, Phe or a neutral His. For Lys, there are two requirements: First, the center of mass of the ring and the Lys sidechain nitrogen must be within 6 Å of one another. Second, the angle between two vectors must be 0 ± 30°. These vectors are (1) the normal vector of the aromatic ring and (2) the vector between the Lys sidechain nitrogen and the aromatic rings center of mass. For Arg the above two requirements need to be met (instead of using the Lys sidechain nitrogen the center of mass of the guanidium group is used instead) and an additional requirement needs to be met. This third requirement is an angle measurement whereby the angle must be either 0° ± 30° (stacked cation-π interaction) or 90° ± 30° (t-shaped cation-π interaction). The angle is between the normal vector

of the guanidium group and the normal vector of the aromatic ring. Definitions for π-π interactions were taken from Zhao *et al.* (15), where a *π* residue is one of Trp, Tyr, Phe or a neutral His. The following requirements need to be met for a *π-π* interaction. First, the distance between the centres of mass of the two aromatic rings had to be ≤ 7.2 Å. Two angles were also determined to classify if the interaction was a *π-π* interaction. The first angle, *θ*, was calculated as the angle between the residue 1's vector and the *π-π* - 90°. The second angle named delta was the angle between the residue 2's vector and the *π-π* - 90°. If both theta and delta were between 0° ± 30° then the interaction would have no *π*-overlap and would not be counted. Otherwise, the interaction was labelled as a *π-π* interaction. A hydrophobic interaction is defined if two hydrophobic residues (Ala, Val, Leu, Ile, Pro, Trp, Phe, Cys, Met) have a non-polar carbon atom within 4 Å of one another. A van der Waals (VDW) interaction is defined as between any two atoms within 4 Å of one another. The program is written so that if an interaction more specific than an VDW interaction is found, only the more specific interaction is shown. Likewise, a salt bridge interaction will not also be defined as a hydrogen bonding interaction even if the criteria for a hydrogen bond has been met.

Interactions were defined at the sidechain/mainchain level between a pair of residues, meaning multiple interactions could be identified between a single pair of residues. To analyze the interactions for both structures and simulations, we developed a python library of definitions for each interaction type to scan against. This library made use of MDAnalysis (17; 18) and is available for download and use as part of the GitHub repository associated with this project: https://www.github.com/kamerlinlab/KIN.

Protein Sequence Analysis

Multiple sequence alignment (MSA) was performed using Modeller v.10.3 (19). Parameters for this procedure were following the Modeller manual. Residue type was used as the alignment feature and maximum gap length was set to 20. The percentage identity matrix was generated using the Clustal Omega webserver (20), providing the previously described multiple sequence alignment.

Predicting Candidate Mutations with KIN

KIN was used to identify candidate single- or double-point mutations with the β-lactamase enzyme TEM1 used as the model system. TEM1 was chosen due the existence of deep mutational scanning data, in which the fitness value of almost all single point mutations in this enzyme was determined (21). The normalized (against wild-type TEM1) fitness values for each mutation were taken from the Supporting Information of ref. (21) without modification. Potential mutations of TEM1 were found by identifying alternative residue combinations in other β-lactamases that form a contact using a pair of residues present in TEM1. Due to the experimental data available, only single point mutations were used in the comparison. To perform filtering based on the distance from the active site, the minimum heavy atom distance between every residue in the crystal structure of TEM1 to the catalytic residues in the active site which are S70 and E166, using *E. coli* numbering (PDB ID:1BTL (22)). For each contact, the residue with the smallest distance to the active site was used to define its distance from the active site.

S2. Supplementary Figures

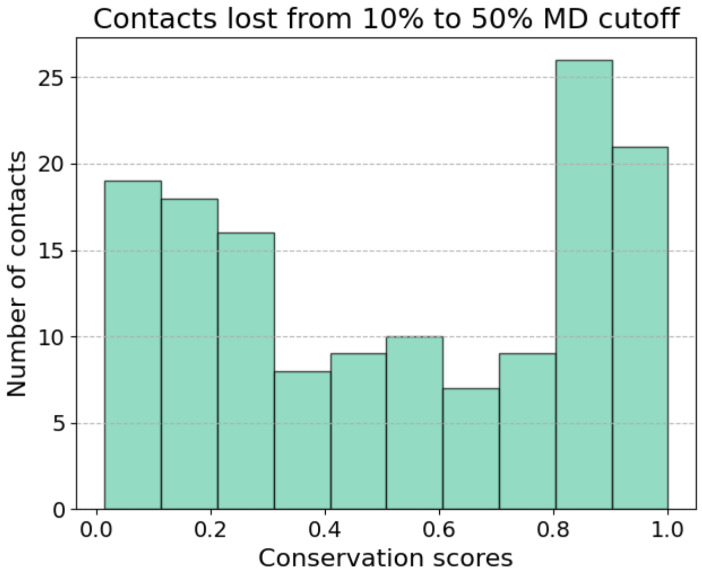

Figure S1. Histogram of the conservation scores removed when changing the MD cutoff filter from 10% to 50% for the β-lactamases studied in this work. The MD cutoff is the minimum percentage of frames a contact has to be present in to be included in the analysis. While some of the interactions that are lost when increasing the cutoff can be discarded as low conservation transient interactions, 32% of lost contacts exhibit strong conservation within a protein family (conserved in >80% of the structures).

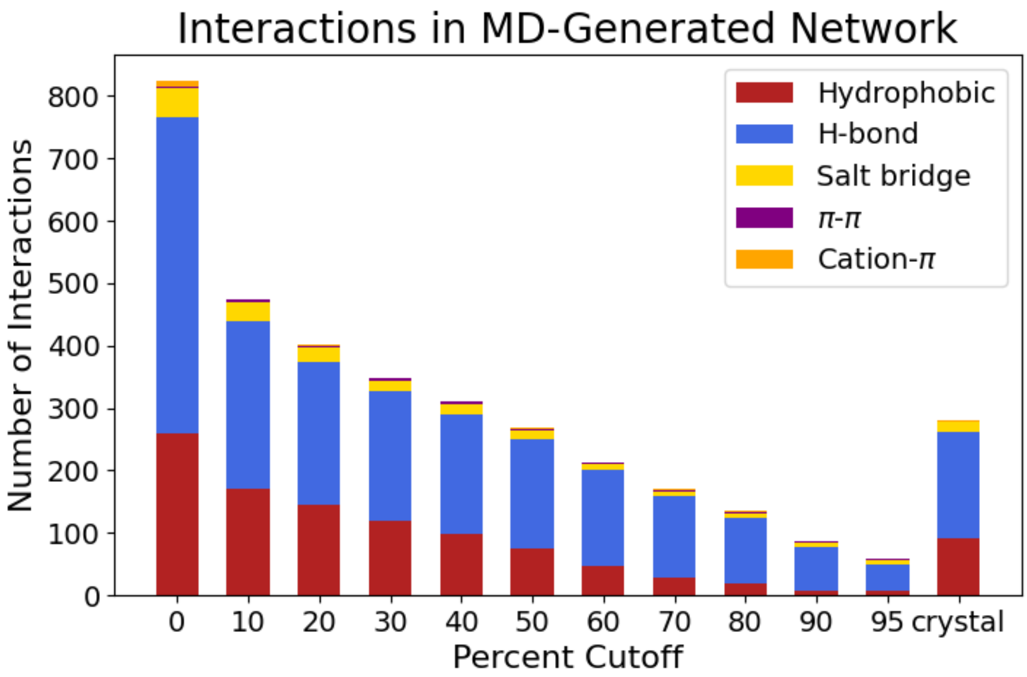

Figure S2. Comparison of the number of contacts identified with different molecular dynamics (MD) simulation retention cut-offs used, with interactions broken down by their interaction type. For reference, the number of interactions obtained when performing analysis using only the crystallographic data (labelled “crystal”) is also shown.

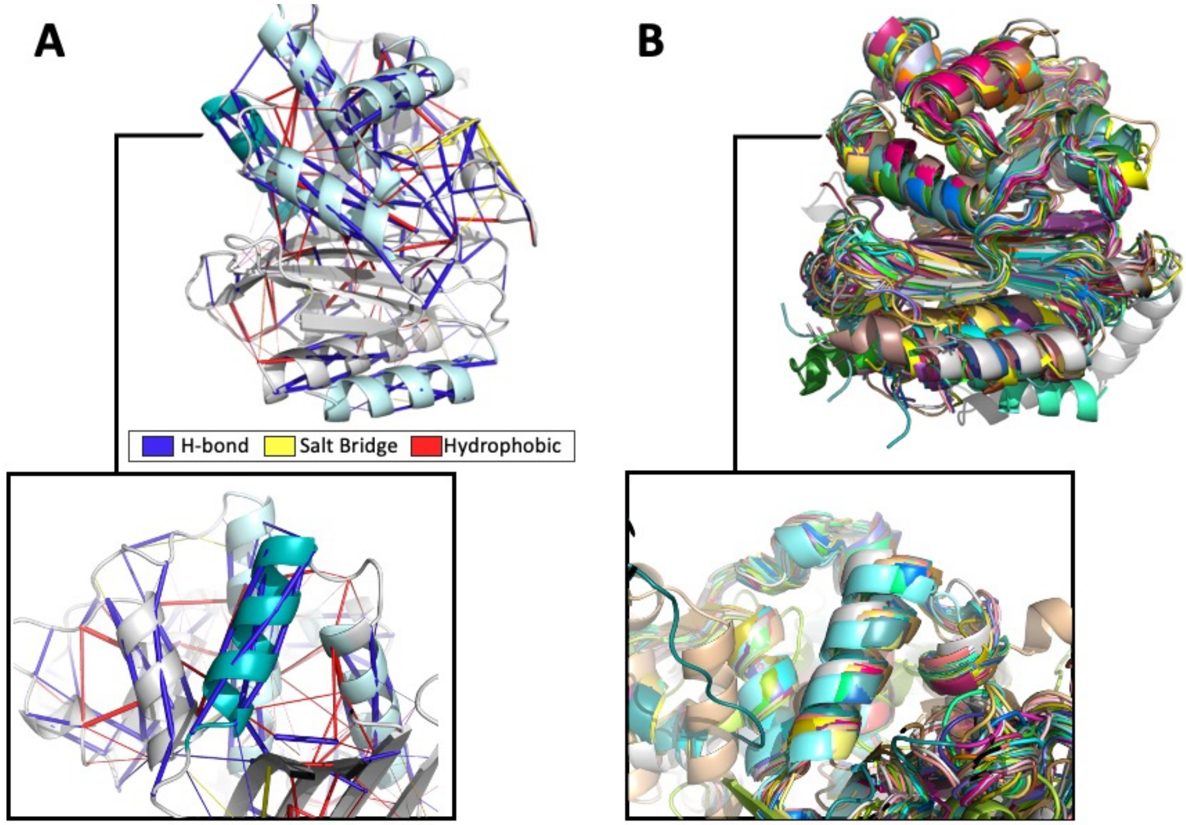

Figure S3. (**A**) A projection of the conserved interaction network generated using KIN on the β-lactamase crystal structure dataset. The projection is performed onto the β-lactamase TEM-1 (PDB 1M40 (23)), with a larger cylinder indicating the interaction is more conserved and the interaction type colored as shown in the legend. (**B**) Structural alignment of 64 out of 69 β-lactamases whose secondary structure elements are highly conserved within the dataset, where 5 of the 69 structures were removed to eliminate domains that lie beyond the preserved core and improve ease of visualization.

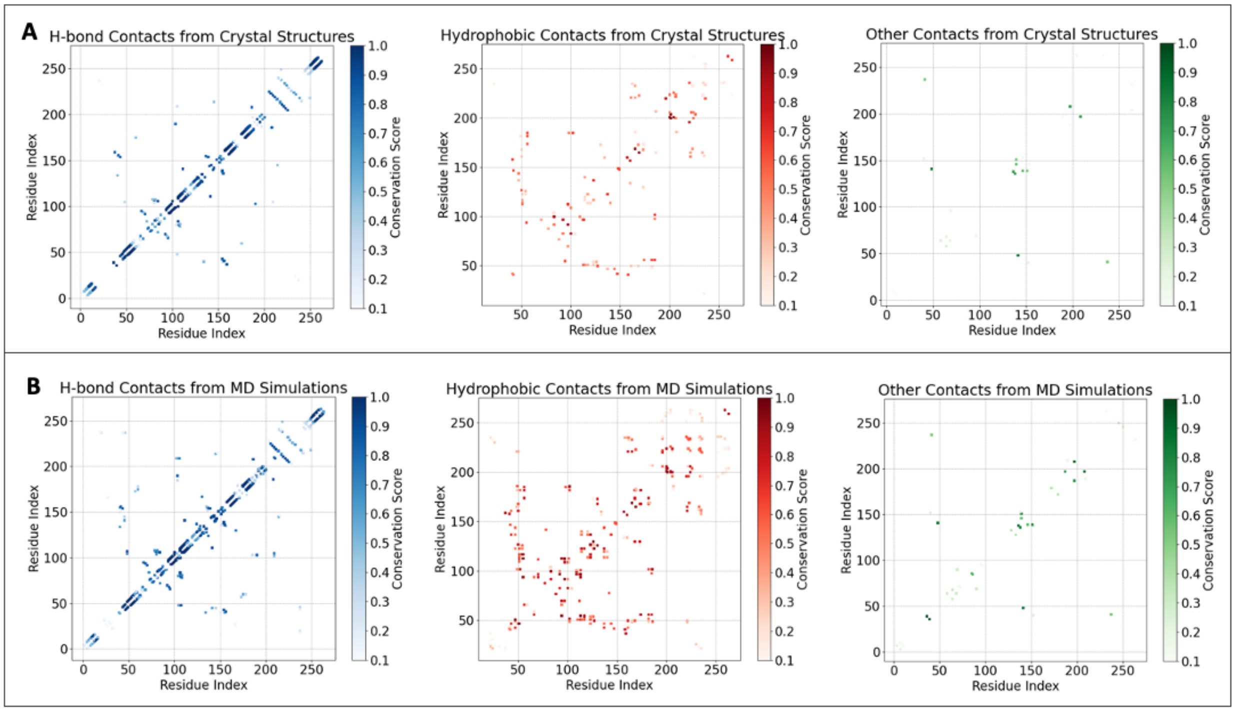

Figure S4. Residue contact maps for the conserved interaction network based on crystal structure (**A**) and MD (**B**) contacts, with a cutoff of 10% used for the MD-based contacts. Contact maps are separated by interaction type: hydrogen bonding, hydrophobic interactions and all other, where other interactions are salt bridges, π- π and cation- π interactions.

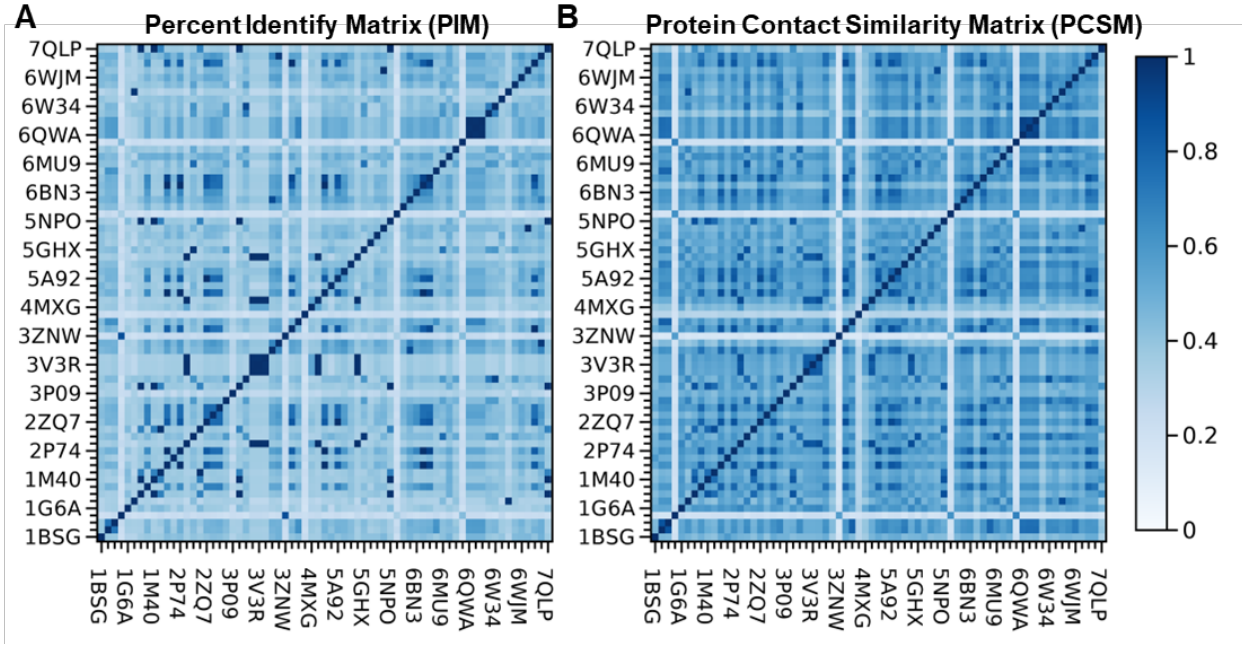

Figure S5. (**A**) The percentage identify matrix (PIM) for the 69 β-lactamase enzyme sequences studied in this manuscript. (**B**) The protein contact similarity matrix (PCSM) determined by our approach which measures how similar each protein's contact network is. For both matrices, the Protein Data Bank (PDB, (1)) IDs are used to label each protein. The PIM values are scaled between 0 and 1 (divided by 100) in order to use the same color scale as the PCSM.

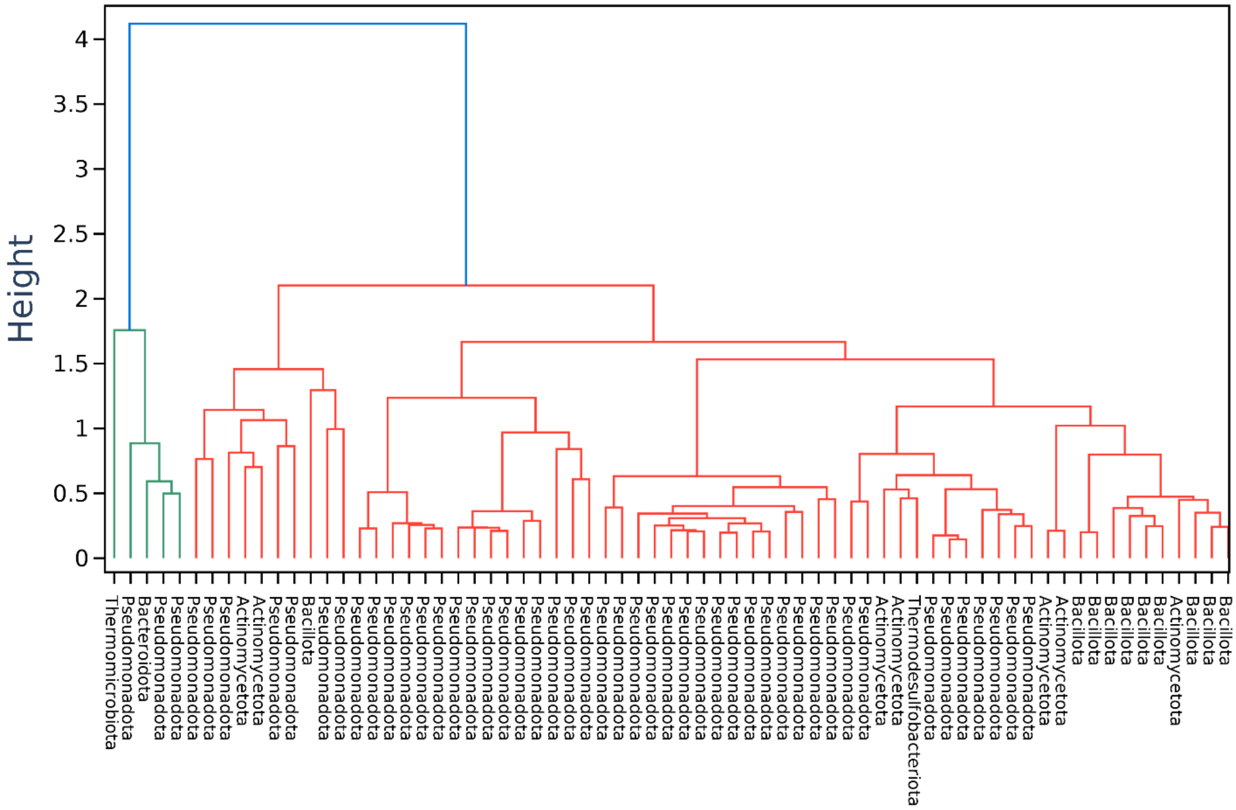

Figure S6. Hierarchal clustering (average linkage) performed on 69 β-lactamase crystal structures using the ratio of shared and unshared contacts between each protein structure pair to create a similarity matrix as input for the clustering. This figure is the same as that shown in the main text (Figure 5A) except the labelling of each protein is now done by the type of bacteria each protein comes from. The phylum of each enzyme was obtained from the UniProtKB (24; 25) database using the UniProt ID associated with each β-lactamase's PDB ID (1).

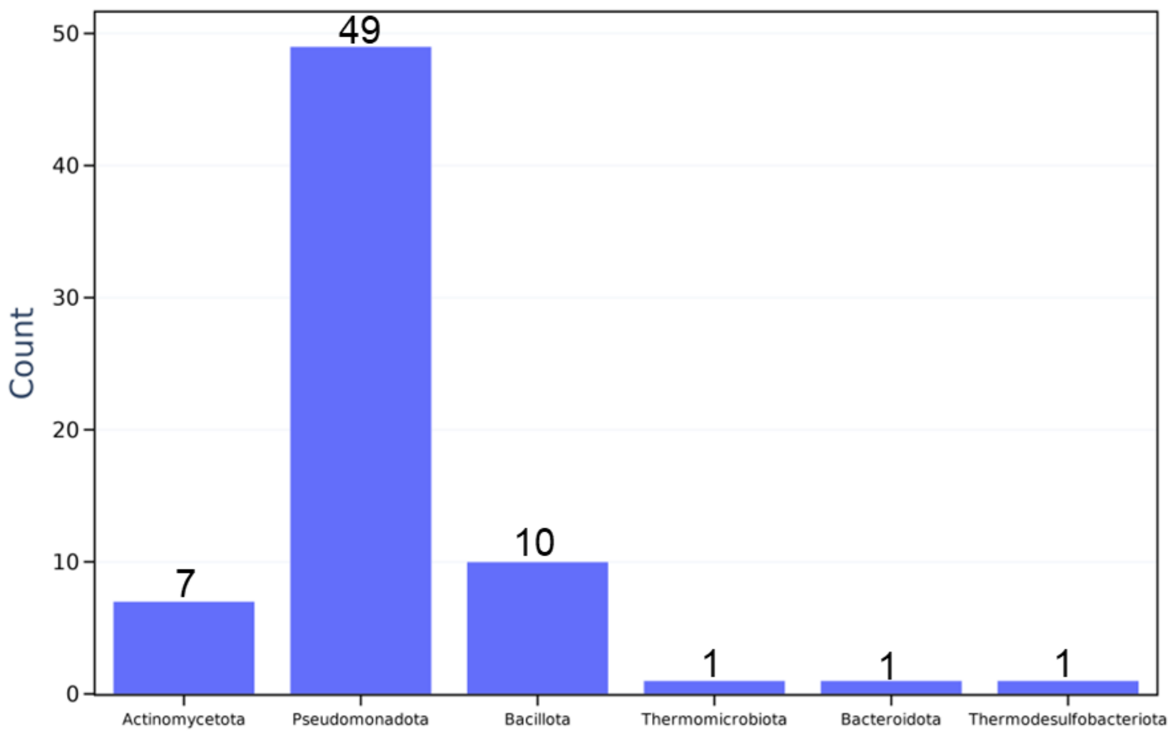

Figure S7. Distribution into phyla of the 69 β-lactamases studied in this manuscript, plotted as a histogram. The phylum enzyme is associated with was obtained from the UniProtKB (24; 25) database using the UniProt ID associated with each β-lactamase's PDB ID (1).

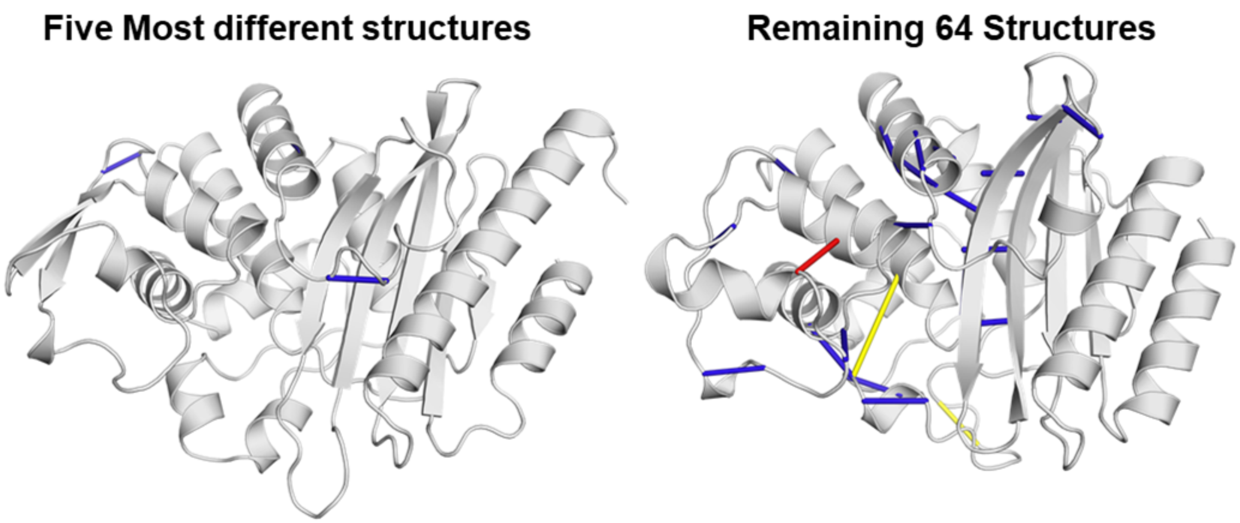

Figure S8. Structural representation of the most different interactions between the five most different β-lactamase structures and the remaining 64 β-lactamases. The five most different β-lactamases were obtained from the hierarchical clustering performed which is shown in the main text, Figure 5. For the representative structures of each cluster above, contacts where the difference in conservation between the clusters was greater than 80% were projected onto the structures (the contact is projected onto the cluster with the higher conservation). Representative structures were selected based on their ability to represent as many of the contacts as possible (see Supplementary Methods). PDB IDs 1E25 (26) and 1HTZ (27) were used to represent the five most different and remaining 64 clusters respectively.

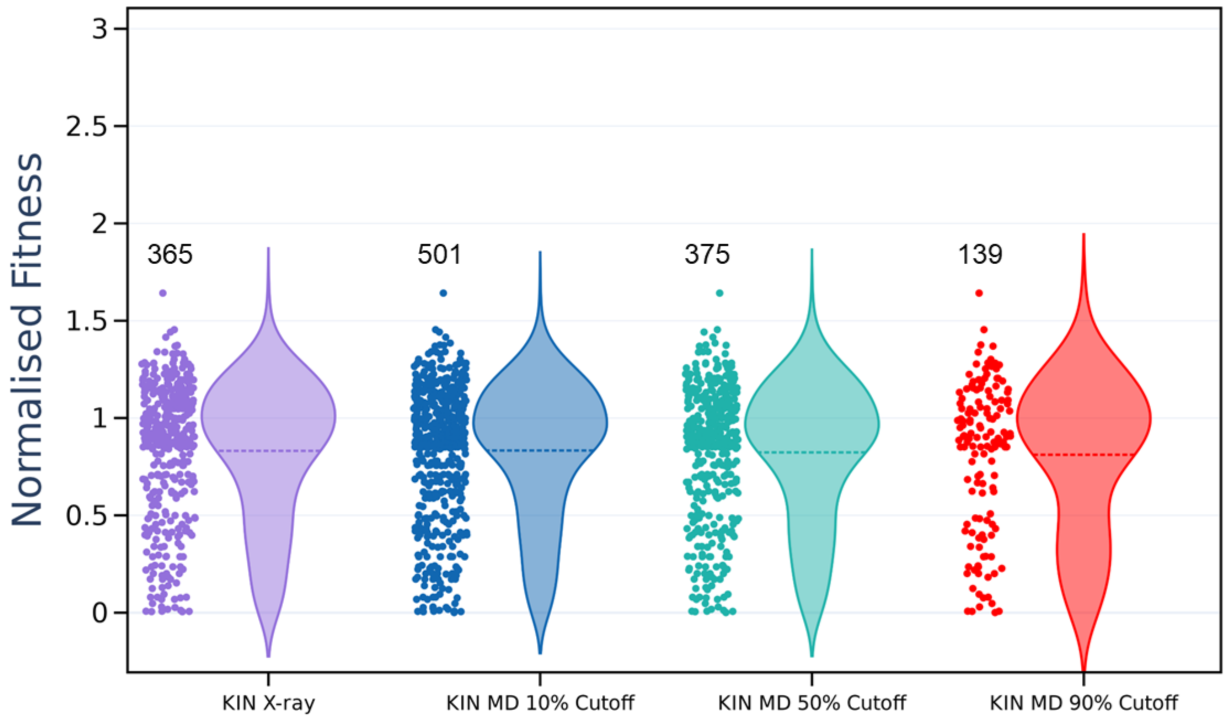

Figure S9. Comparisons of the fitness scores obtained for the single point mutations of TEM1 using different MD simulation cutoffs. Strip and violin plots are shown for each case, with the number of mutations shown above the strip plot and the average value for each condition indicated by a dotted line on the violin plot. The first violin plot is obtained using our approach with just the X-ray structures. The 3 subsequent approaches utilize the MD simulation data with different cut-offs applied to define if a contact is considered present. For example, a 10% cutoff would mean that a contact has to be present in at least 10% of frames for it to be included.

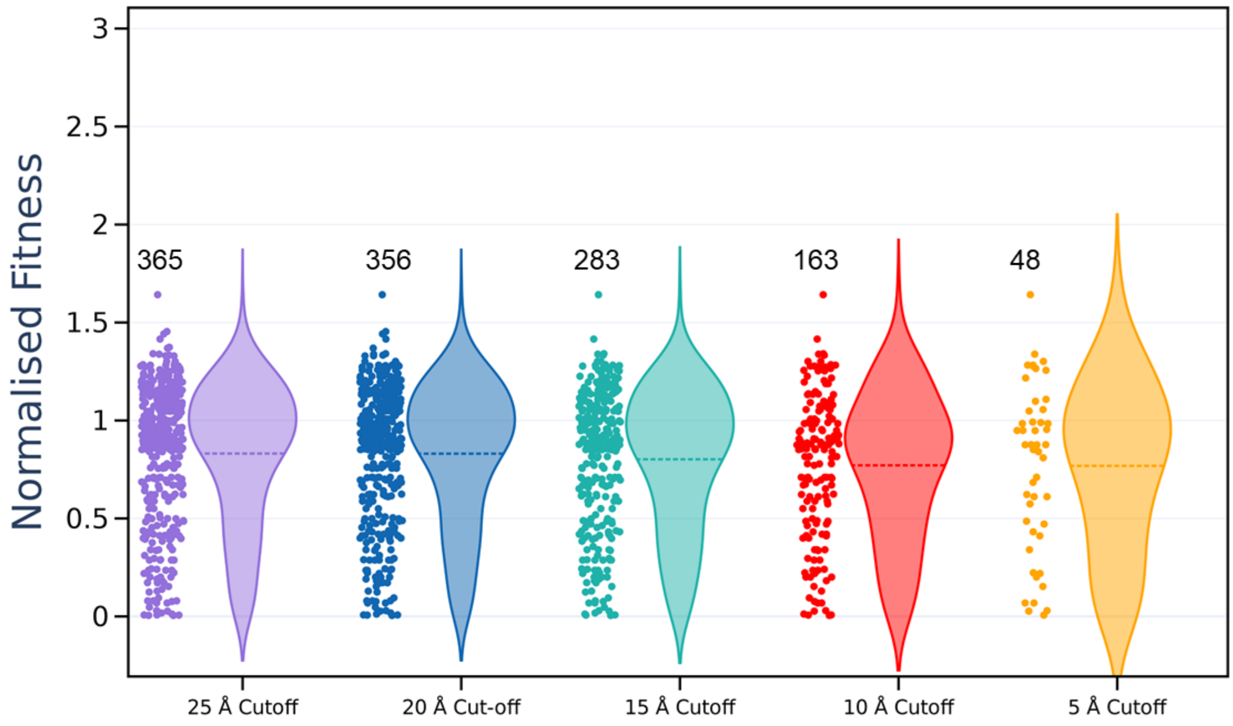

Figure S10. Comparison of the normalized fitness values of mutants selected by KIN using different distance cutoffs from the TEM1 active site. Strip and violin plots are shown for each case, with the number of mutations shown above the strip plot and the average value for each condition indicated by a dotted line on the violin plot. In all cases, the mutations selected here come from crystal structure analysis. The distance of each mutation to the active site is based on the minimum heavy atom distance of each contact to the two catalytic residues: S70 and E166 (*E. coli* numbering, based on PDB ID:1BTL (22)) that make up the active site. We note that at a 25 Å distance cut-off, all residues are included in our analysis.

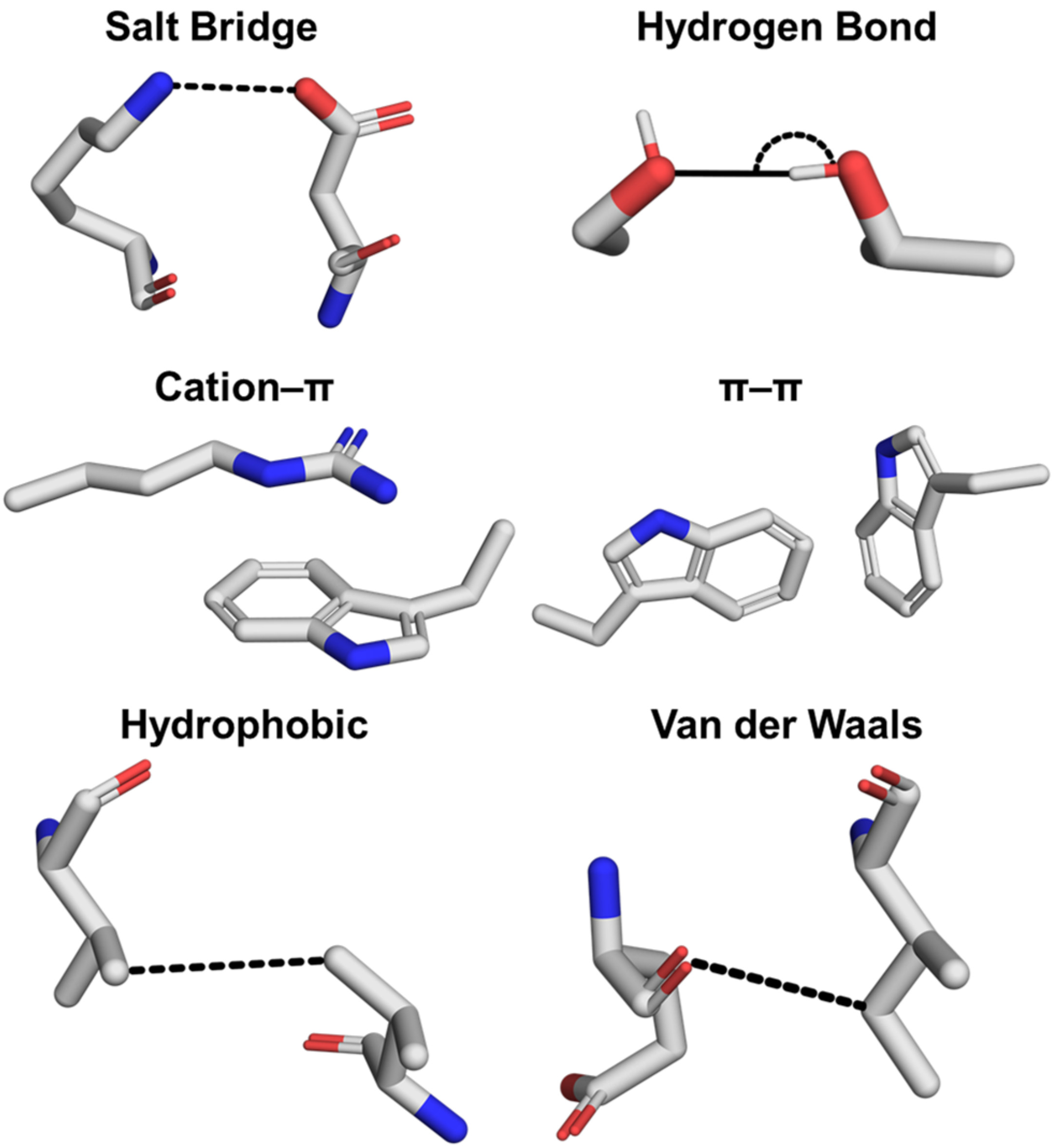

Figure S11. Non-covalent interaction types that can be identified through KIN. This enumeration is also included in the KIN GitHub repository https://www.github.com/kamerlinlab/KIN. The exact distance and angle criteria used to define each interaction type is provided in the Supplementary Methods. For demonstrative purposes, a "stacked" cation-π interaction is shown, however our program will also capture the other type of cation- π interaction ("t-shaped") as described by Infield *et al*. (16). Likewise, whilst a "t-shaped" π-π stacking interaction is shown in the figure above, "face to face" and "offset" π-π stacking interactions can also be identified, see Zhao *et al*. (15), for the definitions of these interaction types.

S3. Supplementary References

1. Berman HM, Westbrook J, Feng Z, Gilliland G, Bhat TN, Weissig H, Shindyalov IN, Bourne PE (2000) The Protein Data Bank. Nucleic Acids Res 28:235-242.

2. Naas T, Oueslati S, Bonnin RA, Dabos ML, Zavala A, Dortet L, Retailleau P, Iorga BI (2017) Beta-Lactamase Database (BLDB) – Structure and Function. J Enzyme Inhib Med Chem 32:917-919.

3. Webb B, Šali A (2016) Comparative Protein Structure Modeling Using Modeller. Curr Protoc Bioinformat 54:5.6.1-5.6.37.

4. Kather I, Jakob RP, Dobbek H, Schmid FX (2008) Increased Folding Stability of TEM-1 β-Lactamase by *In Vitro* Selection. J Mol Biol 383:238-251.

5. Sun T, Nukaga M, Mayama K, Braswell EH, Knox JR (2009) Comparison of β-Lactamases of Classes A and D: 1.5-Å Crystallographic Structure of the Class D OXA-1 Oxacillinase. Prot Sci 12:82-91.

6. Chen VB, Arendall WB, Headd JJ, Keedy DA, Immormino RM, Kapral GJ, Murray LW, Richardson JS, Richardson DC (2010) MolProbity: All-Atom Structure Validation for Macromolecular Crystallography. Acta Crystallogr Sect D Biol Crystallogr 66:12-21.

7. Søndergaard CR, Olsson MHM, Rostkowski M, Jensen JH (2011) Improved Treatment of Ligands and Coupling Effects in Empirical Calculation and Rationalization of pK_a Values. J Chem Theory Comput 7:2284-2295.

8. Case DA, Belfon K, Ben-Shalom IY, Brozell SR, Cerutti DS, Cheatham III TE, Cruzeiro VWD, Darden TA, Duke RE, Giambasu G, Gilson MK, Gohlke H, Goetz AW, Harris R, Izadi S, Izmailov SA, Kasavajhala K, Kovalenko A, Krasny R, Kurtzman T, Lee TS, LeGrand S, Li P, Lin C, Liu J, Luchko T, Luo R, Man V, Merz KM, Miao Y, Mikhailovskii O, Monard G,

Nguyen H, Onufriev A, Pan F, Pantano S, Qi R, Roe DR, Roitberg A, Sagui C, Schott-Verdugo S, Shen J, Simmerling CL, Skyrnnikov NR, Smith J, Swails J, Walker RC, Wang J, Wilson L, Wolf RM, Wu X, Xiong Y, Xue Y, York DM, Kollman PA. AMBER 2020. (2020). University of California, San Francisco.

9. Maier JA, Martinez C, Kasavajhala K, Wickstrom L, Hauser KE, Simmerling C (2015) FF14SB: Improving the Accuracy of Protein Side Chain and Backbone Parameters from FF99SB. J Chem Theory Comput 11:3696-3713.

10. Jorgensen WL, Chandrasekhar J, Madura JD, Impey RW, Klein ML (1983) Comparison of Simple Potential Functions for Simulating Liquid Water. J Chem Phys 79:926-935.

11. Ryckaert J-P, Ciccotti G, Berendsen HJC (1977) Numerical Integration of the Cartesian Equations of Motion of a System with Constraints: Molecular Dynamics of *n*-Alkanes. J Comput Phys 23:327-341.

12. Berendsen HJC, Postma JPM, van Gunsteren WF, DiNola A, Haak JR (1984) Molecular Dynamics with Coupling to an External Bath. J Chem Phys 81:3684-3690.

13. Darden T, York D, Pedersen L (1993) Particle Mesh Ewald: An $N\cdot\log(N)$ Method for Ewald Sums in Large Systems. J Chem Phys 98:10089-10092.

14. Roe DR, Cheatham TE (2013) PTRAJ and CPPTRAJ: Software for Processing and Analysis of Molecular Dynamics Trajectory Data. J Chem Theory Comput 9:3084-3095.

15. Zhao Y, Li J, Gu H, Wei D, Xu Y-C, Fu W, Yu Z (2015) Conformational Preferences of π–π Stacking Between Ligand and Protein, Analysis Derived from Crystal Structure Data Geometric Preference of π–π Interaction. Interdiscip Sci 7:211-220.

16. Infield DT, Rasouli A, Galles GD, Chipot C, Tajkhorshid E, Ahern CA (2021) Cation-π Interactions and their Functional Roles in Membrane Proteins. J Mol Biol 433:167035.

17. Michaud-Agrawal N, Denning EJ, Woolf TB, Beckstein O (2011) MDAnalysis: A Toolkit for the Analysis of Molecular Dynamics Simulations. J Comp Chem 32:2319-2327.

18. Gowers R, Linke M, Barnoud J, Reddy T, Melo M, Seyler S, Domanski J, Dotson D, Buchoux S, Kenney I, Beckstein O (2016) MDAnalysis: A Python Package for the Rapid Analysis of Molecular Dynamics Simulations. Proc 15th Python Sci Conf.

19. Šali A, Blundell TL (1993) Comparative Protein Modeling by Satisfaction of Spatial Restraints. J Mol Biol 234:779-815.

20. Sievers F, Wilm A, Dineen D, Gibson TJ, Karplus K, Li W, Lopez R, McWilliam H, Remmert M, Söding J, Thompson JD, Higgins DG (2011) Fast, Scalable Generation of High-Quality Protein Multiple Sequence Alignments Using Clustal Omega. Mol Syst Biol 7:539.

21. Firnberg E, Labonte JW, Gray JJ, Ostermeier M (2014) A Comprehensive, High-Resolution Map of a Gene's Fitness Landscape. Mol Biol Evol 31:1581-1592.

22. Jelsch C, Mourey L, Masson J-M, Samama J-P (1993) Crystal Structure of *Escherichia coli* TEM1 β-Lactamase at 1.8 Å Resolution. Proteins 16:364-383.

23. Minasov G, Wang X, Shoichet BK (2002) An Ultrahigh Resolution Structure of TEM-1 β-Lactamase Suggests a Role for Glu166 as the General Base in Acylation. J Am Chem Soc 19:5333-5340.

24. Boutet E, Lieberherr D, Tognolli M, Schneider M, Bairoch A (2007) UniProtKB/Swiss-Prott. Methods Mol Biol 406:89-112.

25. The Uniprot Consortium (2018) UniProt: The Universal Protein Knowledgebase. Nucelic Acids Res 46:2699.

26. Tranier S, Bouthors A-T, Maveyraud L, Guillet V, Sougakoff W, Samama J-P (2000) The High Resolution Crystal Structure for Class A β-Lactamase PER-1 Reveals the Bases for Its Increase in Breadth of Activity. J Biol Chem 275:28075-28082.

27. Orencia MC, Yoon JS, Ness JE, Stemmer WPC, Stevens RC (2001) Predicting the Emergence of Antibiotic Resistance By Directed Evolution and Structural Analysis. Nat Struct Biol 8:238-242.
